# Metformin attenuates synergic effect of diabetes mellitus and *Helicobacter pylori* infection on gastric cancer cells proliferation by suppressing PTEN expression

**DOI:** 10.1111/jcmm.15967

**Published:** 2021-03-24

**Authors:** Huibin Lu, Xinwei Han, Jianzhuang Ren, Kewei Ren, Zongming Li, Quanhui Zhang

**Affiliations:** ^1^ Department of Interventional Radiology The First Affiliated Hospital of Zhengzhou University Zhengzhou China

**Keywords:** DM, GC, *H pylori*, metformin, methylation, PTEN

## Abstract

It has been reported that CagA of *Helicobacter pylori* reduced PTEN expression by enhancing its promoter methylation. Furthermore, diabetes mellitus (DM) may also promote the methylation status of PTEN, a tumour suppressor gene in gastric cancer (GC). It is intriguing to explore whether DM may strengthen the tumorigenic effect of *H pylori* (HP) by promoting the methylation of PTEN promoter and whether the administration of metformin may reduce the risk of GC by suppressing the methylation of PTEN promoter. In this study, we enrolled 107 GC patients and grouped them as HP(−)DM(−) group, HP(+)DM(−) group and HP(+)DM(+) group. Bisulphite sequencing PCR evaluated methylation of PTEN promoter. Quantitative real‐time PCR, immunohistochemistry and Western blot, immunofluorescence, flow cytometry and MTT assay were performed accordingly. DNA methylation of PTEN promoter was synergistically enhanced in HP(+)DM(+) patients, and the expression of PTEN was suppressed in HP(+)DM(+) patients. Cell apoptosis was decreased in HP(+)DM(+) group. Metformin showed an apparent effect on restoring CagA‐induced elevation of PTEN promoter methylation, thus attenuating the PTEN expression. The reduced PTEN level led to increased proliferation and inhibited apoptosis of HGC‐27 cells. In this study, we collected GC tumour tissues from GC patients with or without DM/HP to compare their PTEN methylation and expression while testing the effect of metformin on the methylation of PTEN promoter. In summary, our study suggested that DM could strengthen the tumorigenic effect of HP by promoting the PTEN promoter methylation, while metformin reduces GC risk by suppressing PTEN promoter methylation.

## 1 INTRODUCTION

Gastric cancer (GC) is one of highly frequently diagnosed cancers in the world. GC causes the 2nd highest death rate among all types of cancers. The majority of GC cases are gastric carcinoma and gastric antrum cancer, although the occurrence of carcinoma at gastroesophageal junction has been rising steadily.^1^, ^2^, ^3^ Review of the onset of GC showed that the occurrence of GC is slowly increasing among youth adults and in children. Also, the occurrence, death rate and metastasis of GC are pretty high, along with low rate of early diagnosis, good prognosis and 5‐year survival.^4^

The gene of phosphatase and tensin homologue on chromosome 10 (PTEN) is deemed as a tumour suppressor in numerous forms of cancers in human.^5^ PTEN suppresses phosphatidylinositol‐3‐kinase (PI3K) expression through catalysing the elimination of D3 phosphate from phosphatidylinositol (3,4,5)‐ triphosphate (PIP3) to dysregulate the production of phosphatidylinositol 4,5‐bisphosphate (PIP2) and PIP3.^6^, ^7^, ^8^ PTEN is down‐regulated in GC, and the expression of PTEN is also negatively correlated with metastasis in lymph nodes, depth of invasion and the age of GC patients.^9^, ^10^, ^11^ PTEN was shown to promote the cell cycle arrest, apoptosis and metastasis of GC cells.^12^, ^13^ Formerly, Tet1 has been shown to prevent the metastasis and development of GC through PTEN demethylation and its expression. Tet1 reduces the migration, development and invasion of GC through the demethylation of CpG islands in the promoter region of PTEN, thus down‐regulating focal adhesion kinase and AKT activity.^14^

The role of DM in the risk of cancer has been analysed in various meta‐analyses.^15^ Epidemiologic research reviewing the relationship between DM and the risk of GC has generated contradictory results.^16^, ^17^ It was shown that PTEN promoter hypomethylation is quite common among Uyghur patients carrying wild‐type 2 diabetes mellitus (T2DM), which might contribute to T2DM pathogenesis. The abnormal methylation of CpG sites in the promoter of PTEN might function as a biomarker for T2DM diagnosis.

*Helicobacter pylori* (*H pylori*) are a global threat that has infected about 4 billion individuals.^18^ *Helicobacter pylori* are the primary reason for peptic ulcer disease, gastritis and GC. Previous research has revealed that the infection by *H pylori* caused an epithelial‐to‐mesenchymal transition of epithelial cells in the stomach.^19^ It was revealed that CagA considerably lowered the PTEN and Tet1 expression. Furthermore, it was uncovered that CagA lowered PTEN expression through enhancing its own methylation, which was dramatically decreased by Tet1.^20^ Metformin is a compound extracted from Galega officinalis and has been utilized for years in medical treatment to type 2 diabetes mellitus (T2DM).^21^ It was shown that metformin dramatically reduces AKT‐dependent phosphorylation.^22^

CagA of *H pylori* reduces the expression of PTEN by enhancing its promoter methylation.^20^ Furthermore, DM also promotes PTEN methylation.^23^ Giving the fact that DM and HP infection synergistically elevate the risk of GC and the ability of metformin to suppress the methylation of PTEN, we hypothesized that DM may strengthen the tumorigenic effect of HP by promoting PTEN promoter methylation, and the administration of metformin may reduce the risk of GC by suppressing PTEN promoter methylation.^23^, ^24^, ^25^ In this study, we collected GC tumour tissues from GC patients with or without DM/HP to compare their PTEN methylation and expression while testing the effect of Metformin on the methylation of PTEN promoter.

## 2 MATERIALS AND METHODS

### 2.1 Human subjects sample collection

In this study, we enrolled 107 patients with gastric cancer in the First Affiliated Hospital of Zhengzhou University and divided them into three groups according to their status of HP (*H Pylori*) infection and Diabetes mellitus, that is (a) HP(−) DM(−) group (N = 39), (b) HP(+) DM(−) group, (N = 32) and (c) HP(+) DM(+) group, (N = 36). The gastric cancer and HP infection were diagnosed based on the clinical and pathological evidences and the DM were diagnosed following the instructions of American Association of Clinical Endocrinologists.^26^ Those who were diagnosed with other type of cancers, renal dysfunction, liver failure, heart failure or those who take metformin to treat DM were excluded from this study. Gastric cancer stages were classified according to the Guidelines for Stomach Cancer Staging by American Cancer Society. The information of participants, including their age, gender, smoking history, drinking history, staging of gastric cancer and location of gastric cancer, was collected and compared among different groups. The ethical committee of The First Affiliated Hospital of Zhengzhou University has approved the protocol of this study, and all methods were performed in accordance with the last vision of the Declaration of Helsinki. Written informed consent was obtained from all patients before the study.

### 2.2 Bisulphite sequencing

We used bisulphite sequencing to determine the level of DNA methylation in the promoter of the PTEN gene. In brief, genomic DNA in the samples was isolated by making use of a QIAamp DNA Mini assay kit (Qiagen) in accordance with the routine assay protocol provided by the kit manufacturer. Then, bisulphite conversion was carried out via adding 5 mol/L of salt sodium bisulphite to each 1.8 µg of the DNA sample. A universal primer free of CpG was made use of for the amplification of both demethylated and methylated promoter of the PTEN gene at 55.0°C annealing temperature. The product of PCR was then quantitatively evaluated through using DHPLC on a WAVE DNA Fragment Analysis System in accordance with the routine assay protocol provided by the manufacturer.

### 2.3 RNA isolation and real‐time PCR

Real‐time PCR was done to measure the expression of PTEN mRNA in collected samples. In brief, total RNA in each sample was isolated by utilizing a PureLink RNA Mini assay kit (Thermo Fisher Scientific) in accordance with the routine assay protocol provided by the kit manufacturer. Then, cDNA was generated from isolated total RNA by making use of a PrimeScript RT Reagent assay kit (Thermo Fisher Scientific) in accordance with the routine assay protocol provided by the kit manufacturer. In the next step, the real‐time PCR was carried out on an iCycler real‐time PCR instrument (Bio‐Rad Laboratories) by utilizing an iQSYBR Green master kit (Bio‐Rad Laboratories) in accordance with the routine assay protocol provided by the kit manufacturer. The ΔΔCt method^27^ was used to determine the relative expression of PTEN (forward primer: 5′‐ TGAGTTCCCTCAGCCGTTACCT‐3′; reverse primer: 5′‐GAGGTTTCCTCTGGTCCTGGTA‐3′) in each sample, with housekeeping gene GAPDH (forward primer: 5′‐CAAAGTTGTCATGGATGACC‐3′; reverse primer: 5′‐CCATGGAGAAGGCTGGGG‐3′) serving as the internal reference.

### 2.4 Cell culture and transfection

HGC‐27 cells, an origin human gastric carcinoma cell line, were bought from American Type Culture Collection (ATCC) and maintained in an RPMI 1640 medium (Gibco, Thermo Fisher Scientific) added with 10% of foetal bovine serum (Gibco, Thermo Fisher Scientific) and a suitable amount of penicillin and streptomycin (Gibco, Thermo Fisher Scientific). The culture was carried out at 37°C in an incubator containing 5% CO_2_. Prior to the experiments, the cells were randomly divided into four groups, that is (a) Untreated group (HGC‐27 cells treated with PBS only); (b) Metformin group (HGC‐27 cells treated with 100 µmol/L of metformin); (c) CagA group (HGC‐27 cells treated with CagA); (d) CagA + Metformin group (HGC‐27 cells pre‐treated with CagA and subsequently treated with 100 µmol/L of metformin). After 48 hours of treatment, the cells were collected for subsequent assays.

### 2.5 MTT cell proliferation assay

The proliferation status of treated cells was measured by utilizing a CellTiter‐Glo MTT assay (Promega) in accordance with the routine assay protocol provided by the kit manufacturer.

### 2.6 Western blot analysis

Collected tissue and cell samples were lysed by using a TRIzol reagent (Invitrogen) in accordance with the routine assay protocol provided by the reagent manufacturer to obtain protein lysate, which was then resolved via electrophoresis by making use of a 10% SDS‐PAGE gel. After transferring the resolved protein samples onto PVDF membranes, the membranes were blocked by making use of 5% skim milk and then incubated overnight at 4°C with anti‐PTEN primary antibody (R&D systems). In the next step, the membranes were washed and further incubated at room temperature for 1 hour with horseradish peroxidase‐labelled secondary antibody. Finally, the relative expression of PTEN protein in each sample was analysed after the protein blot was developed by making use of an Immobilon Western Chemiluminescent HRP Substrate assay kit (EMD Millipore) in accordance with the routine assay protocol provided by the kit manufacturer.

### 2.7 Apoptosis analysis

The status of apoptosis in each collected cell and tissue sample was analysed by using a propidium iodide/annexin V‐FITC apoptosis assay kit (Sigma Aldrich) in accordance with the routine assay protocol provided by the kit manufacturer. The detection of apoptosis was carried out on a FACSCanto II flow cytometer (BD Biosciences) at a 488 nm wavelength.

### 2.8 Immunofluorescence and immunohistochemistry

Immunofluorescence and immunohistochemistry assays were used to detect the expression of PTEN and the status of apoptosis of collected tissue samples, respectively. In brief, for immunohistochemistry assays, the tissue samples were fixed by using 4% of formaldehyde (Sigma Aldrich), deparaffinized and rehydrated using gradient alcohol. In the next step, the sections were stained with anti‐PTEN primary antibody (Abcam) and suitable biotin‐labelled secondary antibody (Thermo Fisher Scientific) before the relative PTEN expression in each sample was evaluated under a Zeiss LSM700 confocal laser‐scanning microscope in accordance with the instruction provided by the manufacturer. For immunofluorescence assays, the status of apoptosis of collected tissue samples was evaluated by using a Vybrant Apoptosis Assay Kit (Thermo Fisher Scientific) in accordance with the routine assay protocol provided by the kit manufacturer.

### 2.9 Statistical analysis

All statistical evaluations were conducted by making use of GraphPad Prism 8.0 for Microsoft Windows (GraphPad, La Jolla, CA). All data were shown as mean ± SEM. The difference between two different groups was evaluated by using a two‐tailed Student's *t* test. A *P* value of <.05 was deemed significant statistically.

## 3 RESULTS

### 3.1 The characteristics of patients

We enrolled 107 gastric cancer patients and divided them into three groups according to their status of HP (*H Pylori*) infection and Diabetes mellitus: (a) HP(−)DM(−)(N = 39), (b) HP(+)DM(−)(N = 32), and (c) HP(+)DM(+)(N = 36). The information of participants was collected and listed in Table 1. Student's *t* test was utilized to perform the statistical comparison and revealed that there was no obvious difference in all above characteristics among the three groups.

**Table 1:** Demographic and clinical characteristics of recruited subjects

Characteristics	HP(−)DM(−) (N = 39)	HP(+)DM(−) (N = 32)	HP(+)DM(+) (N = 36)	*P* value
Age, years	58 ± 0.43	57 ± 0.25	57 ± 0.15	
<60	18 (46.2)	14 (43.8)	18 (50.0)	.279
≥60	21 (53.8)	18 (56.2)	18 (50.0)	
Gender				
Male	30 (76.9)	24 (75.0)	31 (86.1)	.538
Female	9 (23.1)	8 (25.0)	5 (13.9)	
Smoking				
Ever or current	20 (51.3)	18 (56.3)	20 (55.6)	.608
Never	17 (43.6)	13 (40.6)	15 (41.6)	
Missing	2 (5.1)	1 (3.1)	1 (2.8)	
Drinking				
Ever or current	18 (46.2)	17 (53.1)	20 (55.6)	.178
Never	20 (51.2)	14 (43.8)	14 (38.8)	
Missing	1 (2.6)	1 (3.1)	2 (5.6)	
Stage				
Early	15 (38.5)	13 (40.6)	18 (50.0)	.376
Advanced	13 (33.3)	8 (25.0)	8 (22.2)	
Missing	11 (28.2)	11 (34.4)	10 (27.8)	
Location				
Cardia	11 (28.2)	8 (25.0)	10 (27.8)	.882
Non‐cardia	25 (64.1)	23 (71.9)	25 (69.4)	
Missing	3 (7.7))	1 (3.1)	1 (2.8)	

### 3.2 HP infection and Diabetes mellitus synergistically increased the DNA methylation level of PTEN promoter in gastric cancer patients

**FIGURE 1:**
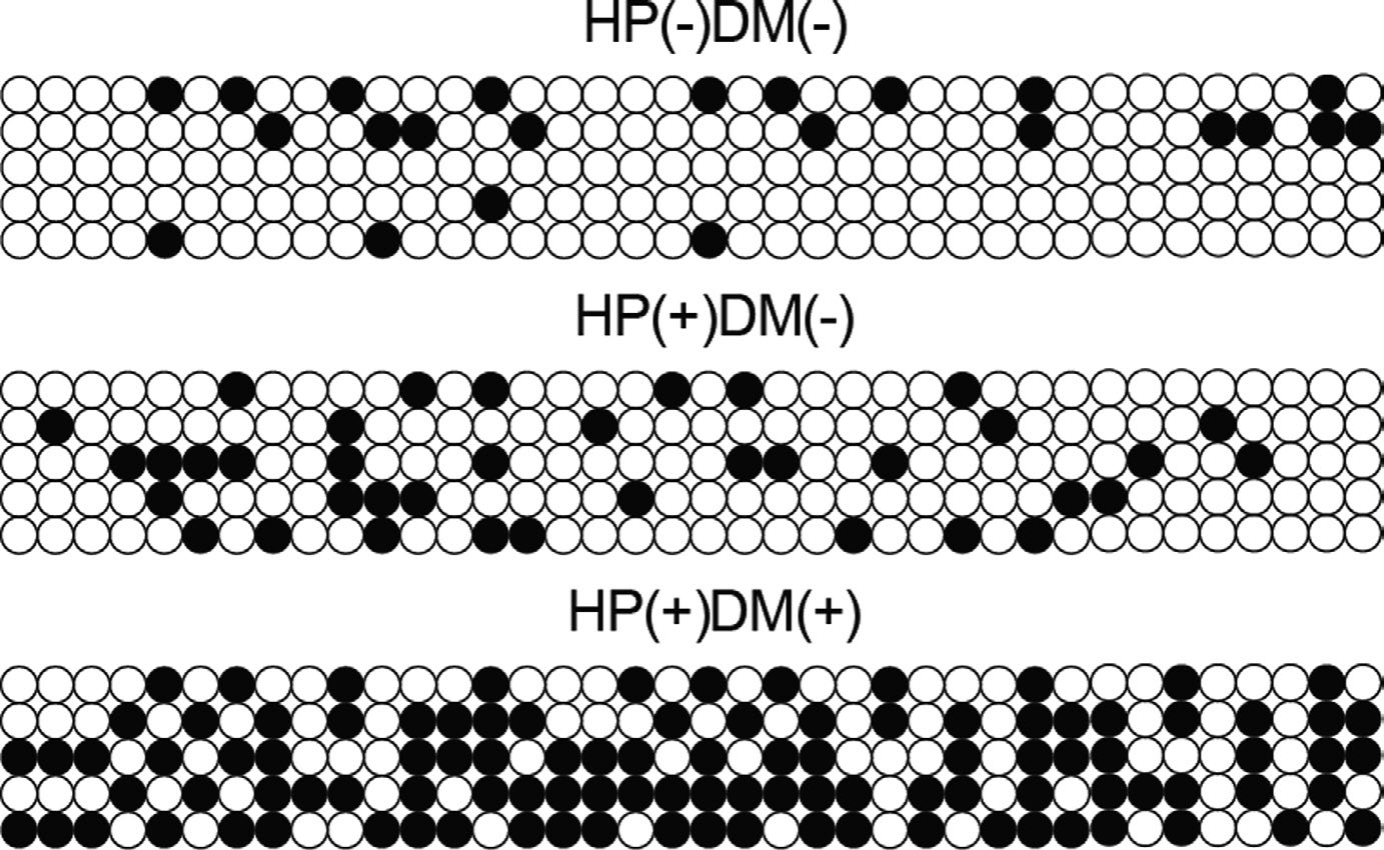
Bisulphite sequencing PCR analysis showed increased DNA methylation of PTEN promoter in HP(−)DM(−), HP(+)DM(−) and HP(+)DM(+) patients with gastric cancer (The white dot denotes unmethylated CpG site and the black dot denotes methylated CpG site)

As PTEN expression was reported to be closely related to the pathogenesis of gastric cancer, and DNA methylation level of PTEN promoter is reversely correlated with PTEN expression, we performed bisulphite sequencing PCR to evaluate the DNA methylation status of PTEN promoter in the three groups of gastric cancer patients. As shown in Figure 1, DNA methylation of PTEN promoter was slightly increased in HP(+)DM(−) patients when compared with HP(−)DM(−) patients. However, DNA methylation of PTEN promoter was remarkably elevated in HP(+)DM(+) patients, indicating that HP infection and diabetes mellitus showed a synergistic effect on promoting the DNA methylation of PTEN promoter in patients with gastric cancer.

### 3.3 HP infection and Diabetes mellitus suppressed the expression of PTEN mRNA and protein in gastric cancer patients in a synergistic manner

**FIGURE 2:**
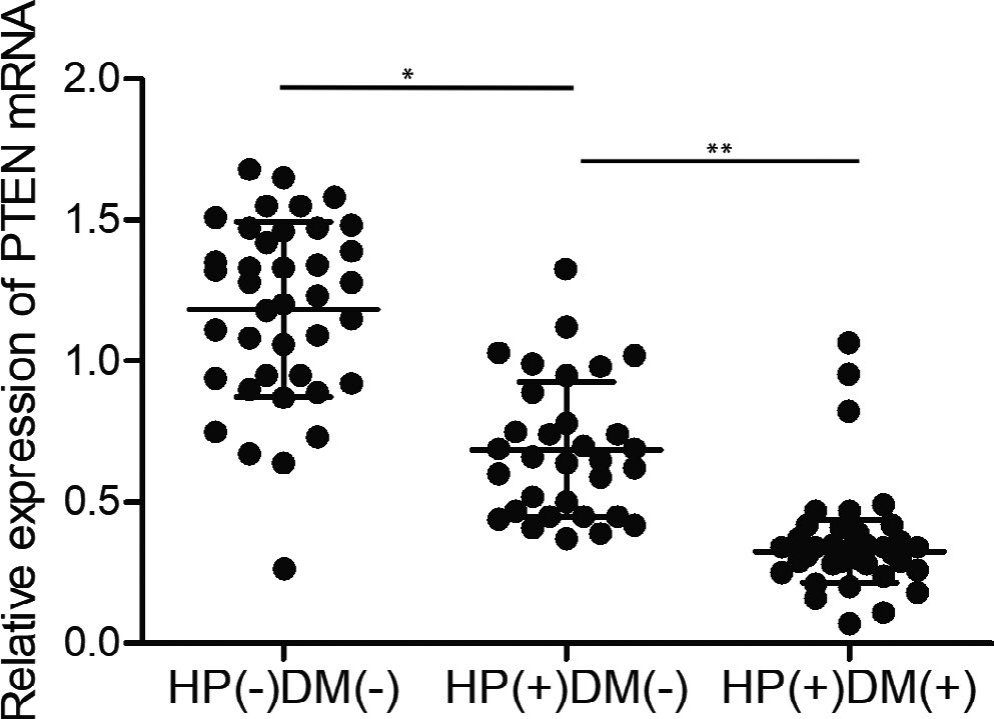
Decreased expression of PTEN mRNA in HP(−)DM(−), HP(+)DM(−) and HP(+)DM(+) patients with gastric cancer (*P value < .05, vs HP(−)DM(−) group; **P value < .05 vs HP(+)DM(−) group)

**FIGURE 3:**
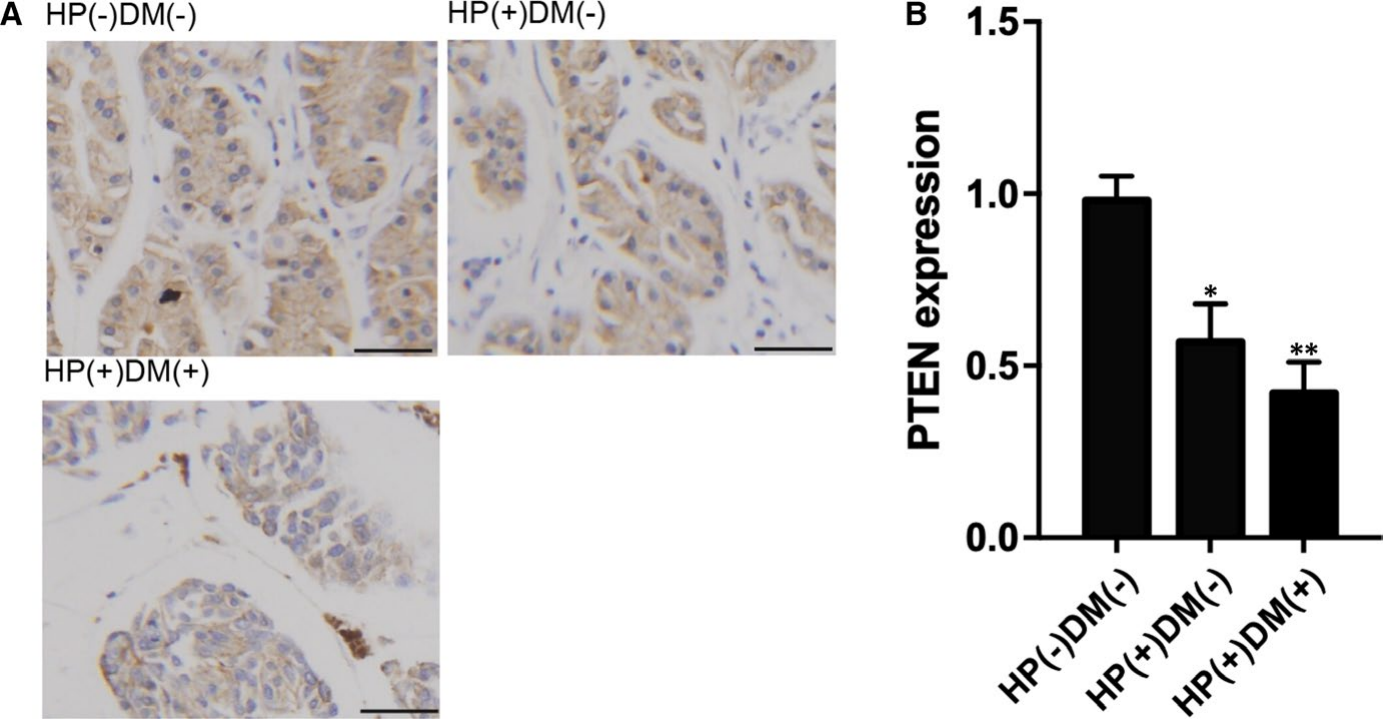
Decreased expression of PTEN protein in HP(−)DM(−), HP(+)DM(−) and HP(+)DM(+) patients with gastric cancer. A, Immunohistochemistry assay of PTEN expression showed lowest PTEN expression in HP(+)DM(+) patients (Scale bar, 10 µm). B, PTEN expression was highest in HP(−)DM(−) patients and lowest in HP(+)DM(+) patients (*P value < .05, vs HP(−)DM(−) group; **P value < .05 vs HP(+)DM(−) group)

It is well known that DNA methylation in the promoter region can repress gene expression. Therefore, we further performed quantitative real‐time PCR and immunohistochemistry assay to examine the PTEN expression in gastric cancer patients in the above three groups. As expected, the expression of PTEN mRNA (Figure 2) and protein (Figure 3) was significantly repressed in HP(+)DM(−) patients in comparison with that in HP(−)DM(−) gastric patients. Moreover, the expression of PTEN mRNA and protein in HP(+)DM(+) gastric cancer patients was more suppressed.

### 3.4 The apoptosis of gastric cancerous tissues was inhibited by HP infection and diabetes mellitus

**FIGURE 4:**
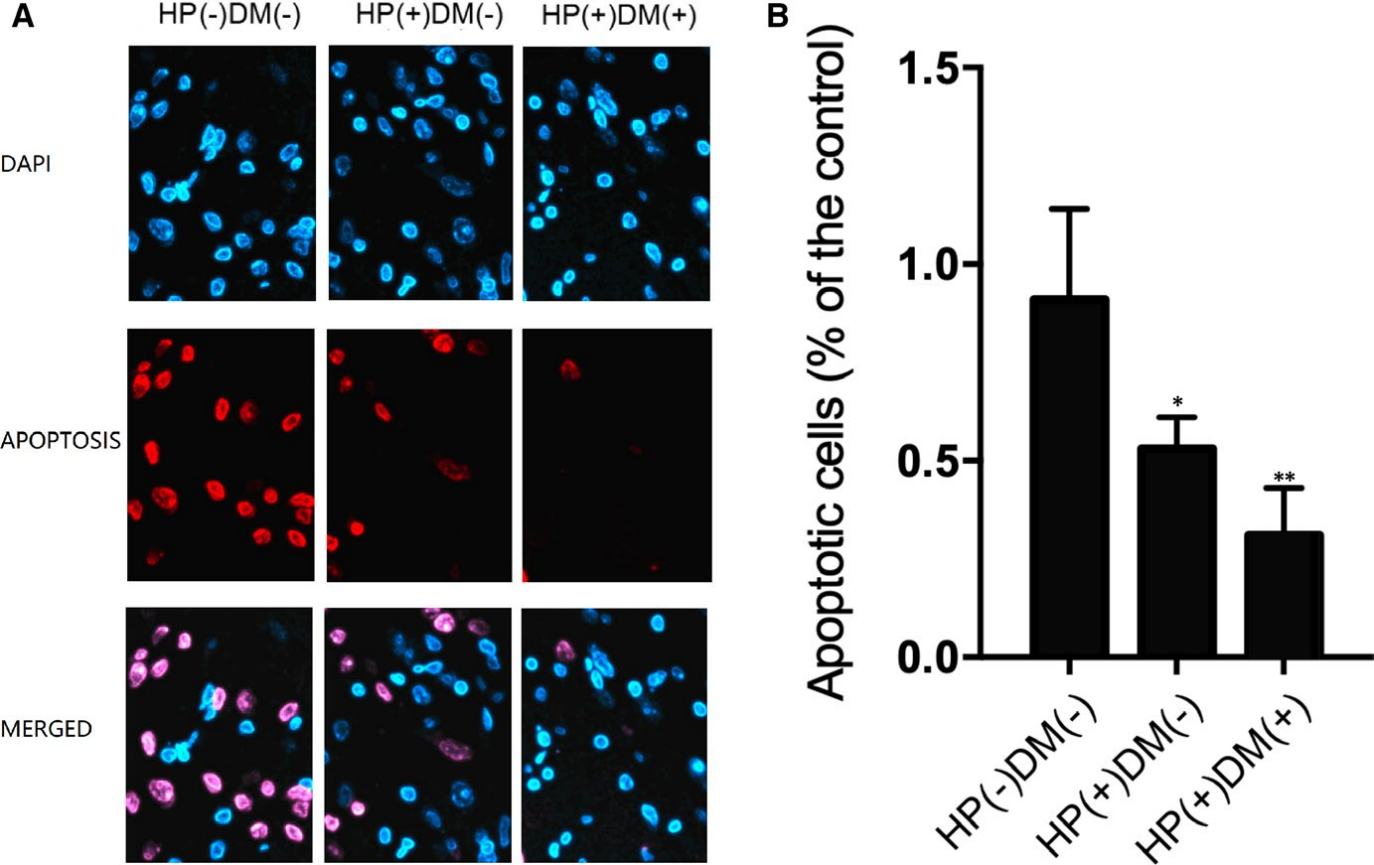
Immunofluorescence analysis showed that the apoptosis of gastric tissues was decreased in HP(−)DM(−), HP(+)DM(−) and HP(+)DM(+) patients with gastric cancer. A, Immunofluorescence analysis of apoptosis of gastric tissues was decreased in HP(−)DM(−), HP(+)DM(−) and HP(+)DM(+) patients with gastric cancer. B, The apoptosis of gastric tissues was decreased in HP(−)DM(−), HP(+)DM(−) and HP(+)DM(+) patients with gastric cancer (*P value < .05, vs HP(−)DM(−) group; **P value < .05 vs HP(+)DM(−) group)

Furthermore, immunofluorescence was carried out to evaluate the apoptotic status of gastric tissues collected from patients in HP(−)DM(−), HP(+)DM(−) and HP(+)DM(+) groups. The proportion of apoptotic gastric tissues was progressively decreased from HP(−)DM(−) group to HP(+)DM(−) and HP(+)DM(+) (Figure 4). These results demonstrated that HP infection and Diabetes mellitus played an antagonistic role in the apoptosis of gastric cancer tissues.

### 3.5 Metformin treatment attenuated CagA‐induced increase of DNA methylation of PTEN promoter in HGC‐27 cells

**FIGURE 5:**
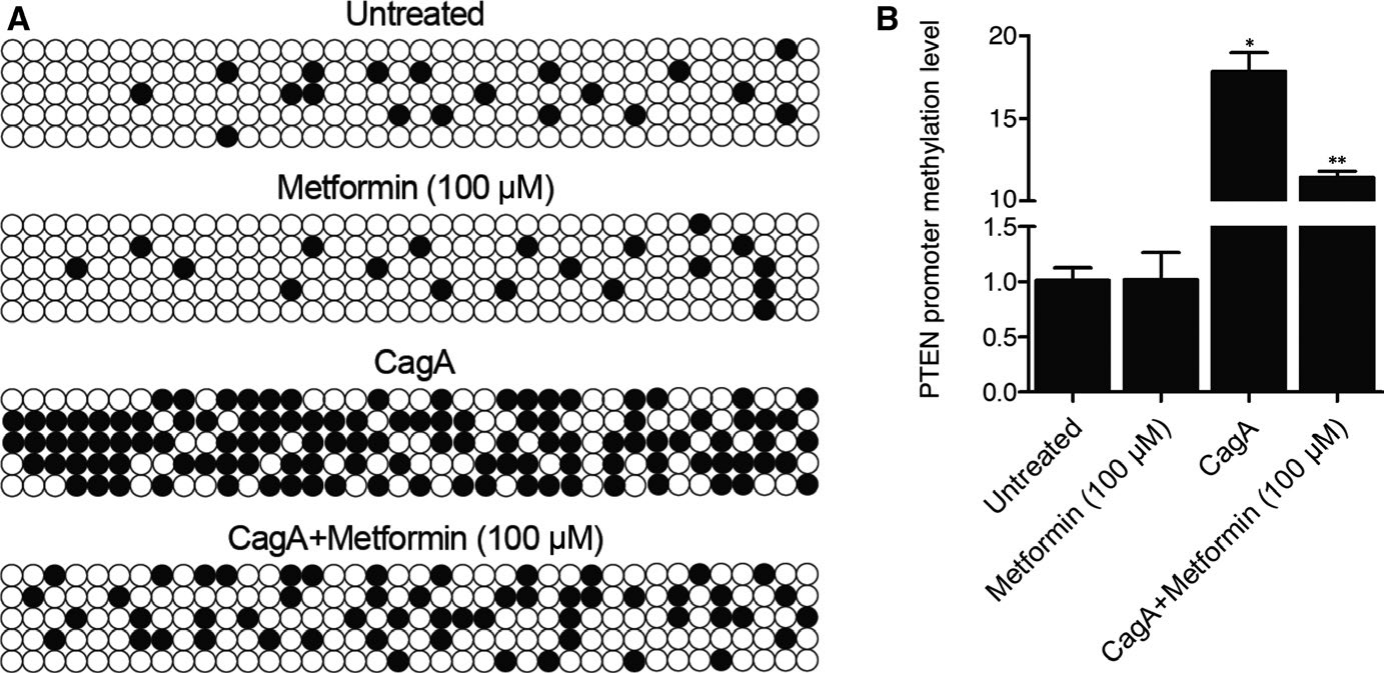
Metformin treatment reversed CagA‐induced hypermethylation of PTEN promoter. A, Bisulphite sequencing PCR analysis demonstrated that metformin treatment decreased CagA‐induced up‐regulation of PTEN mRNA expression in HGC‐27 cells. B, Metformin treatment decreased CagA‐induced up‐regulation of PTEN mRNA expression in HGC‐27 cells (*P value < .05 vs Untreated group; **P value < .05, vs CagA group)

Bisulphite sequencing PCR was carried out to measure the DNA methylation of PTEN promoter in HGC‐27 cells treated under different conditions. We found that DNA methylation of PTEN promoter was obviously elevated in HGC‐27 cells treated with CagA when compared with that in the control cells, whereas no obvious difference was observed in metformin‐treated HGC‐27 cells. However, the DNA methylation of PTEN promoter in HGC‐27 cells enhanced by CagA treatment was effectively reduced by metformin treatment (Figure 5).

### 3.6 Metformin treatment reversed CagA‐induced down‐regulation of PTEN expression in HGC‐27 cells

**FIGURE 6:**
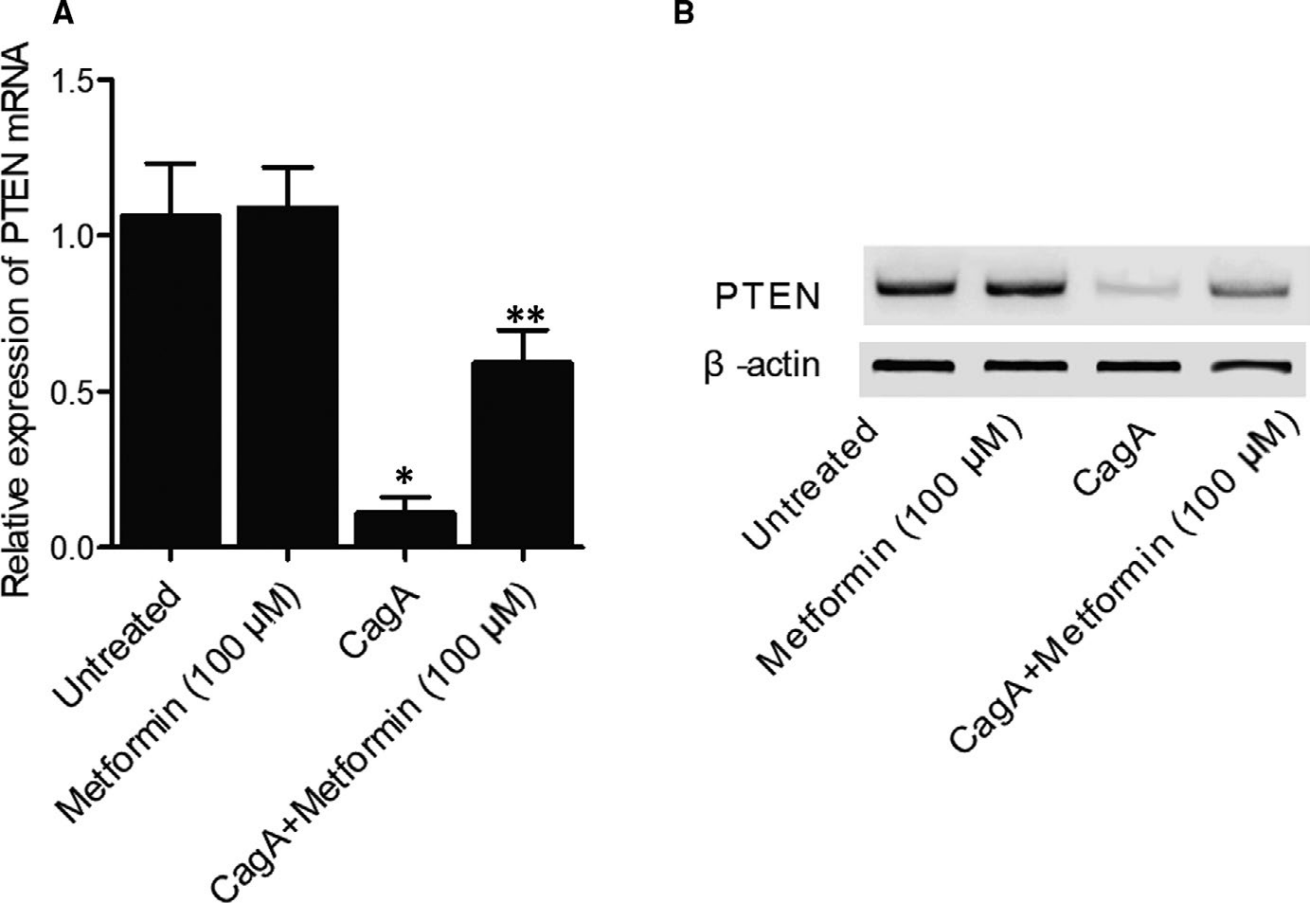
Metformin reversed CagA‐induced decrease of PTEN expression in HGC‐27 cells. A, CagA‐induced decrease of PTEN mRNA expression was effectively reversed by metformin treatment (*P value < .05 vs Untreated group; **P value < .05, vs CagA group). B, Western blot assay indicated that CagA‐induced decrease of PTEN protein expression was effectively reversed by metformin treatment

Quantitative real‐time PCR and Western blot were used to analyse the expression of PTEN mRNA and protein in HGC‐27 cells treated under different conditions. The expression of PTEN mRNA (Figure 6A) and protein (Figure 6B) in HGC‐27 cells was significantly suppressed by CagA treatment, while Metformin treatment showed no obvious effect on the PTEN mRNA expression in untreated HGC‐27 cells. However, metformin treatment effectively reversed CagA‐induced down‐regulation of PTEN mRNA and protein in HGC‐27 cells.

### 3.7 Metformin treatment reversed CagA‐induced dysregulation of proliferation and apoptosis of HGC‐27 cells

**FIGURE 7:**
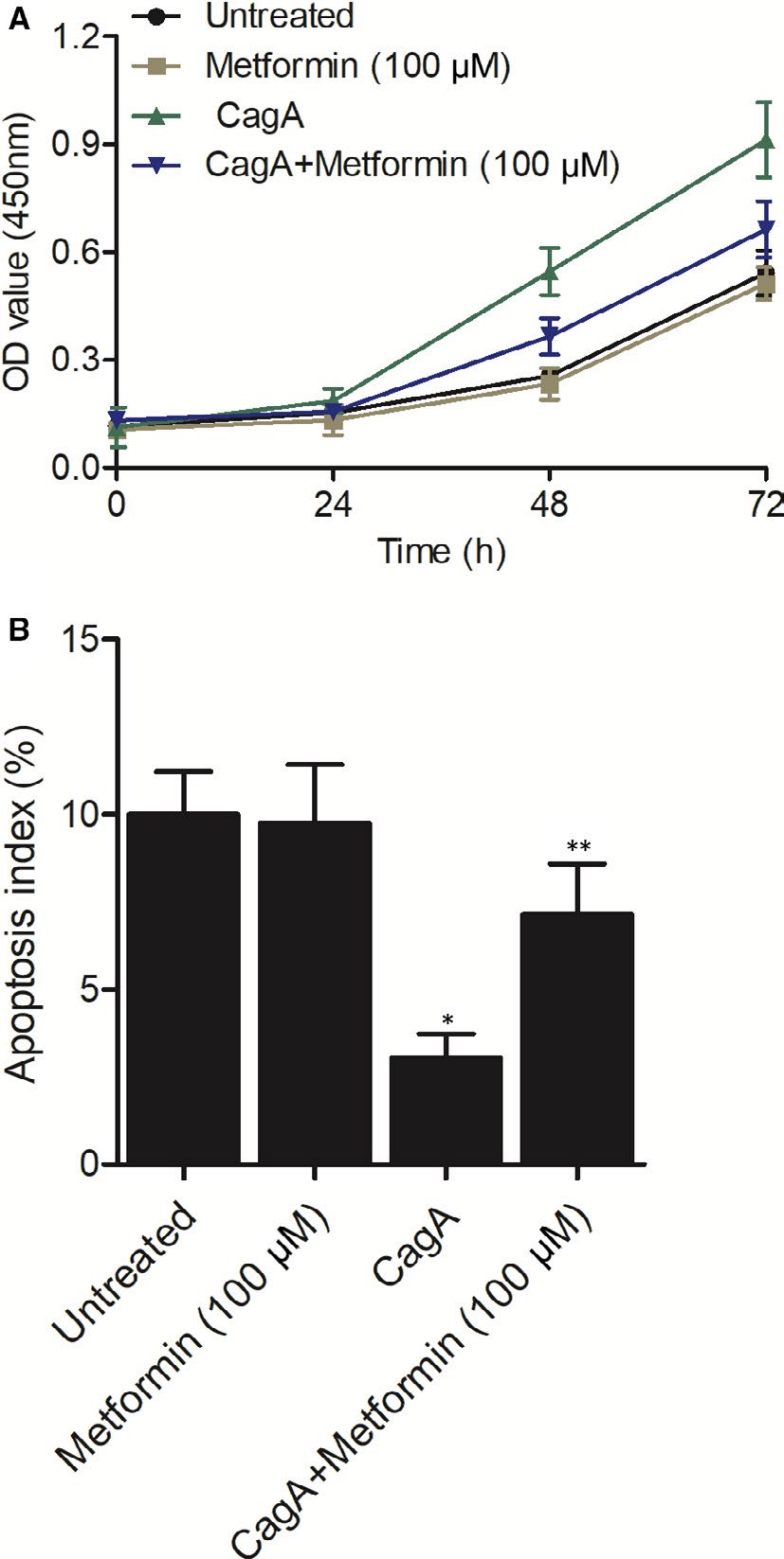
Metformin reversed CagA‐induced dysregulation of proliferation and apoptosis of HGC‐27 cells. A, CagA‐induced increase of HGC‐27 cell proliferation was effectively reversed by metformin treatment. B, CagA‐induced attenuation of HGC‐27 cell apoptosis was effectively reversed by metformin treatment (*P value < .05 vs Untreated group; **P value < .05, vs CagA group)

Finally, we performed MTT assay and flow cytometry to evaluate the proliferation and apoptosis of HGC‐27 cells treated under different conditions. CagA treatment notably increased the proliferation of HGC‐27 cells. Further treatment with metformin significantly decreased CagA‐induced elevation of HGC‐27 cell proliferation (Figure 7A). On the contrary, CagA treatment remarkably suppressed the apoptosis of HGC‐27 cells. Metformin treatment effectively obstructed CagA‐induced decrease of HGC‐27 cell apoptosis (Figure 7B).

## 4 DISCUSSION

Metformin is well known for its anti‐diabetic role.^28^ It was also shown that metformin reduced the risk of cancer in patients with T2DM.^29^, ^30^ The role of metformin in suppressing the proliferation of GC cells is related to its role in blocking cell cycles, which could explain why metformin decreased tumour size in mice with xenograft GC tissues.^31^, ^32^ It was shown that the use of metformin significantly decreased the risk of infection by *H pylori* dose dependently.^33^ In this study, we recruited 107 gastric cancer patients and divided them into three groups according to their status of HP infection and DM to explore the molecular mechanism underlying gastric cancer pathogenesis. We found that HP infection and DM effectively increased the DNA methylation of PTEN promoter in GC patients.

PTEN acts as a crucial tumour suppressor in the carcinogenesis of a wide array of cancers.^34^ PTEN can degrade the derivatives of PI3K through dephosphorylating position 30 of PIP3 and PIP2, subsequently reducing AKT phosphorylation and inducing apoptosis.^35^, ^36^ It was shown that the methylation of PTEN promoter can be used as a prognostic marker of survival.^37^ It was illustrated that GC cells transfected by PTEN‐shRNA NC displayed considerably down‐regulated expression of PTEN, thus substantially enhancing the expression of b‐catenin. In addition, IFC and Western blotting results showed that PTEN down‐regulation and b‐catenin up‐regulation in GC tissues triggered the phosphorylation of p‐AKT and p‐GSK‐3b in GC tissues. In this study, we carried out qPCR and IHC to measure the expression of PTEN mRNA and protein in GC patients in the three groups. HP infection and DM evidently decreased the expression of PTEN mRNA and protein in GC patients. In addition, we used immunofluorescence assays to analyse the apoptosis of GC tissue samples collected from GC patients in the three groups. HP infection and DM notably attenuated the apoptosis of GC tissue samples.

*Helicobacter pylori* infection is one of the most significant dangers for stomach cancers.^38^ In a research on 114 GC patients, gastric adenocarcinoma was shown as accounting for 90% in all patients. In *H pylori*, the expression of a 120 kDa protein called cytotoxin associated gene A (CagA) was shown to be related to some of the essential features of *H pylori* to interact with kinases in host cells to trigger tyrosine phosphorylation.^39^, ^40^, ^41^ So far, 4 distinguish EPIYA motifs have been found, that is EPIYA‐A to EPIYA‐D.^42^, ^43^ It was likewise revealed that CagA dramatically lowered the expression levels of PTEN, APOBEC3A, Tet1, APOBEC3C and APOBEC3F in GC tissues. Furthermore, CagA lowered the expression levels of PTEN through boosting its levels of methylation, which was substantially blocked through up‐regulation of Tet1.^20^ It was additionally found that infection with *H pylori* strains positive for CagA expression triggers Akt signalling in the epithelial tissues in the stomach, thus attenuating cell apoptosis while promoting cell survival.^44^, ^45^, ^46^ The activation of Akt is a common event in chemotherapy treatment of GC, indicating a significant role of Akt in causing apoptosis resistance.^47^, ^48^ Previous results presented that etoposide boosted the phosphorylation of Akt while minimizing cell survival via the induction of GC apoptosis.^49^

It was shown that the abnormal methylation in PTEN promoter was substantially reduced in Uyghur subjects suffering from mild cases of T2DM. It was further shown that the hypomethylation in PTEN promoter was pretty common in T2DM subjects, indicating that PTEN might contribute to T2DM pathogenesis among Uyghur people.^23^ A previous research illustrated that the enhanced PTEN expression in adipose tissues and muscular tissues of T2DM rodents might exert a significant effect on insulin resistance in T2DM.^50^ Zhu et al proposed that PTEN controls the production of extracellular matrix in kidneys through activating Akt while enhancing CTGF in T2DM.^51^, ^52^ In this study, we tested the therapeutic effect of Metformin on HGC‐27 cells stimulated by CagA. We found that Metformin treatment could effectively restore CagA‐induced dysregulation of PTEN promoter methylation, PTEN mRNA and protein expression, as well as the proliferation and apoptosis of HGC‐27 cells.

## 5 CONCLUSION

In summary, these findings suggest that the hypermethylation of PTEN promoter is a common event in GC patients with DM and metformin treatment. DM could strengthen the tumorigenic effect of HP by promoting the methylation of PTEN promoter, while the administration of metformin reduces the risk of GC by suppressing the methylation of PTEN promoter.

## CONFLICT OF INTEREST

The authors declare that they have no conflicts of interest.

## AUTHOR CONTRIBUTIONS

**Huibin Lu:** Conceptualization (equal); Formal analysis (equal); Supervision (equal); Writing‐original draft (equal). **Xinwei Han:** Conceptualization (equal); Project administration (equal); Validation (equal); Writing‐review & editing (equal). **Jianzhuang Ren:** Investigation (equal); Methodology (equal); Software (equal); Writing‐original draft (equal). **Kewei Ren:** Resources (equal); Software (equal); Validation (equal); Visualization (equal). **Zongming Li:** Investigation (equal); Validation (equal); Visualization (equal). **Quanhui Zhang:** Investigation (equal); Methodology (equal).

## Data Availability

The data that support the findings of this study are available from the corresponding author upon reasonable request.
